# Etanercept treatment for pediatric toxic epidermal necrolysis induced by deflazacort: a case report and literature review

**DOI:** 10.3389/fimmu.2024.1342898

**Published:** 2024-01-25

**Authors:** Min Song Jeong, Yun Young Choi, Yo Han Ahn, Kyeonghun Lee, Ji Soo Park, Dong In Suh

**Affiliations:** Department of Pediatrics, Seoul National University College of Medicine, Seoul, Republic of Korea

**Keywords:** toxic epidermal necrolysis, Steven Johnson syndrome, etanercept, deflazacort, severe cutaneous adverse reaction, pediatric

## Abstract

Toxic epidermal necrolysis (TEN) is a life-threatening mucocutaneous disorder commonly caused by drugs. TEN is often treated with corticosteroids, intravenous immunoglobulin (IVIG), or cyclosporine; however, the efficacy of these treatments is controversial. Etanercept (a TNF-α antagonist) was proven to decrease skin-healing time in a randomized clinical trial. Herein, we report the case of a 44-month-old boy who developed TEN due to deflazacort as the probable culprit drug and was successfully treated with etanercept. The patient presented to the emergency department complaining of erythematous maculopapular rashes and vesicles all over the face and body, with vesicles on the hands, feet, and trunk. Symptoms started 4 days before presentation, with edema of the upper lip, which progressed to erythematous macules over the body. He was started on deflazacort for nephrotic syndrome 21 days before the visit. Approximately 20% of the body surface area (BSA) was covered by vesicular lesions. Under the diagnosis of Steven Johnson syndrome/TEN, deflazacort was discontinued, and intravenous dexamethasone (1.5 mg/kg/day), a 5-day course of IVIG (0.4 mg/kg/day), and cyclosporine (3 mg/kg/day) were administered. The lesions seemed to be stationary for 3 days, but on the 6^th^ day of hospitalization, when IVIG was discontinued, the vesicular lesions progressed to approximately 60% of the BSA. Etanercept 0.8 mg/kg was administered subcutaneously. Lesions stopped progressing, and bullous lesions started epithelialization. However, on the 15th day, around 30% of the BSA was still involved; thus, a second dose of etanercept was administered. No acute or sub-acute complications were observed. In conclusion, the use of etanercept in children with TEN that is not controlled with conventional therapy is both effective and safe.

## 1 Introduction

Toxic epidermal necrolysis (TEN) is a life-threatening mucocutaneous disorder often induced by drugs (1). Patients with TEN exhibit over 30% body surface area (BSA) for epidermal detachment, while those with Steven Johnson syndrome (SJS) have less than 10% BSA for skin detachment. Approximately 10%–30% of skin detachments are classified as overlapping SJS-TEN. Flu-like symptoms, such as fever, malaise, poor oral intake, sore throat, and headache, precede the onset of mucocutaneous symptoms, which are atypical targetoid maculopapules that form blisters (2).

TEN is mostly caused by drugs, such as sulfonamide or beta-lactam antibiotics, anti-seizure medications (phenytoin, lamotrigine, and carbamazepine), allopurinol, and nonsteroidal anti-inflammatory drugs (NSAIDs) (3, 4). Other causes include *Mycoplasma pneumoniae*, dengue virus, cytomegalovirus, and the use of contrast agents (2). The probability of the culprit drug is assessed through algorithm of drug causality for epidermal necrolysis (ALDEN), which is based on six parameters: 1) the time interval between the start of the drug and the onset of the reaction (index day), 2) the likelihood that the drug was present on the index day, 3) the presence of a history of adverse effects with the same drug, 4) whether the drug was discontinued as the disease progressed, 5) whether the drug has been documented in previous studies (3, 4), and 6) consideration of alternative possibilities (5).

TEN treatment involves systemic steroids, intravenous immunoglobulin (IVIG), or cyclosporine; however, the efficacy of these treatments is controversial (6). Recent studies showed elevated TNF-α levels in patients with SJS-TEN (7), and etanercept, a TNF-α antagonist, reduced skin-healing time in a randomized clinical trial (8). Herein, we report a case of a 44-month-old boy who developed TEN due to deflazacort as the probable culprit drug and was successfully treated with etanercept.

## 2 Case description

A 44-month-old boy presented to the emergency department with a fever of up to 38.4°C, sore throat, and erythematous maculopapular rashes and vesicles all over the face and body, with vesicles on the hands, feet, and trunk. The rashes and vesicles made the patient complain of severe pain and itching sensation. Symptoms began 4 days prior to presentation, with edema of the upper lip and vesicles in both hands. He was diagnosed with nephrotic syndrome and was taking deflazacort for nephrotic syndrome 21 days prior to the visit. Other complaints included diarrhea and dysuria. Physical examination showed that his eyes were also involved, with swelling of the eyelids, conjunctival injection, and yellowish mucous discharge. Erythematous maculopapular rashes appeared on the cheeks, neck, trunk, arms, penis, and fluid vesicles on both hands and feet. The patient also had a crust on his lips.

Laboratory tests showed a white blood cell count of 12,260 cells/mm^3^ (eosinophil count of 800 cells/mm^3^), hemoglobin level of 15.4 g/dL, and platelet count of 375,000 cells/*mm* ^3^. Serum biochemistry showed blood urea nitrogen (BUN) level of 10 IU/L, creatinine level of 0.48 mg/dL, aspartate aminotransferase (AST)/alanine aminotransferase (ALT) level of 12/27 IU/L, C-reactive protein level of 1.83 mg/dL, and procalcitonin level of 0.206 ng/mL. Blood and urine cultures were negative for *Mycoplasma pneumoniae*, herpes simplex virus, varicella-zoster virus, and human herpes virus antigen.

**Figure 1:**
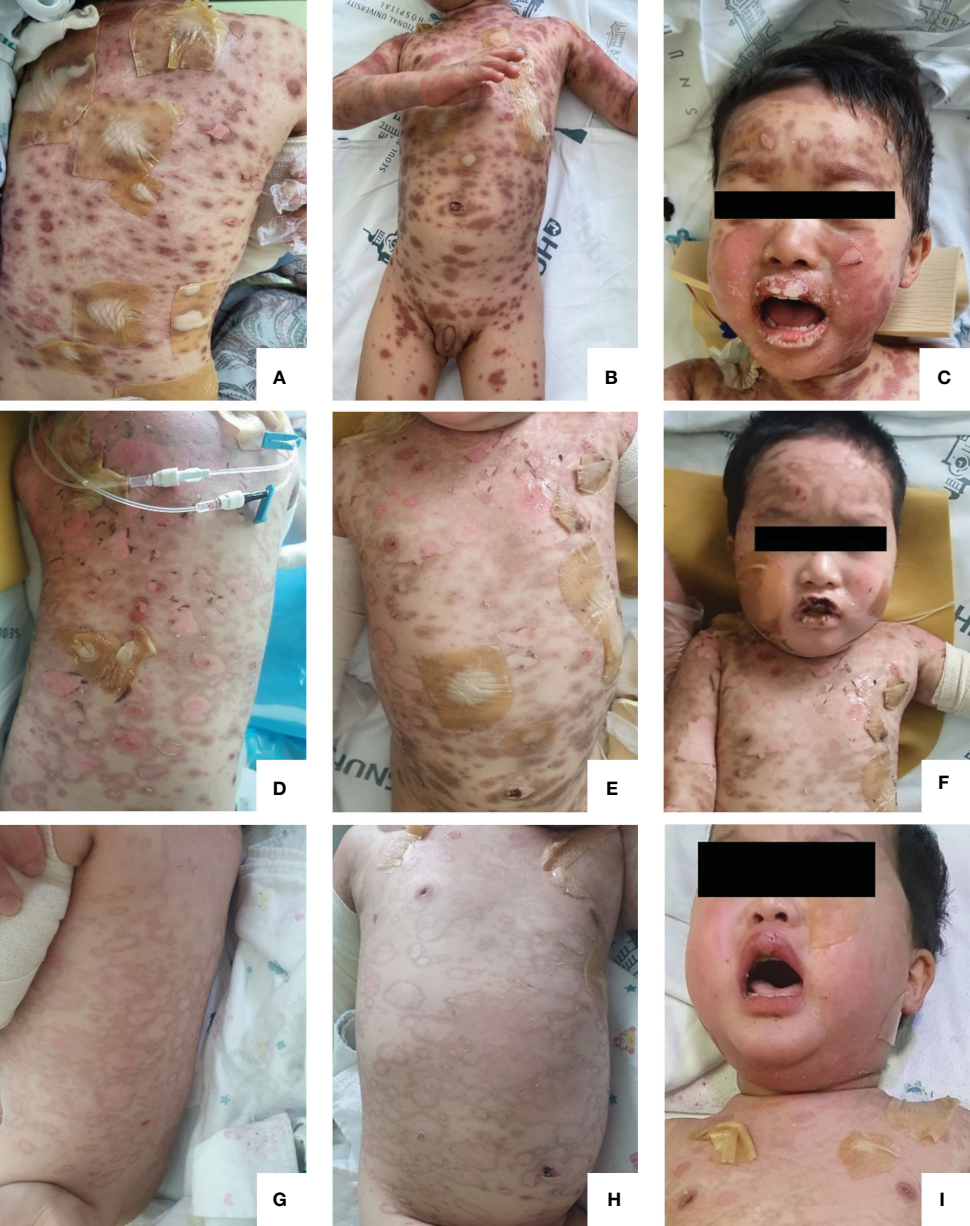
Toxic epidermal necrolysis. **(A–C).** HD#7: Prior to the first etanercept injection, the patient had maculopapular rashes and vesicles throughout the body. **(D–F).** HD#11: After the first dose of etanercept, epithelization began, but some vesicles and bullae remained. **(G–I)**. HD#18: After the second dose of etanercept, the patient’s skin was mostly epithelialized. HD, hospital day.

**Figure 2:**
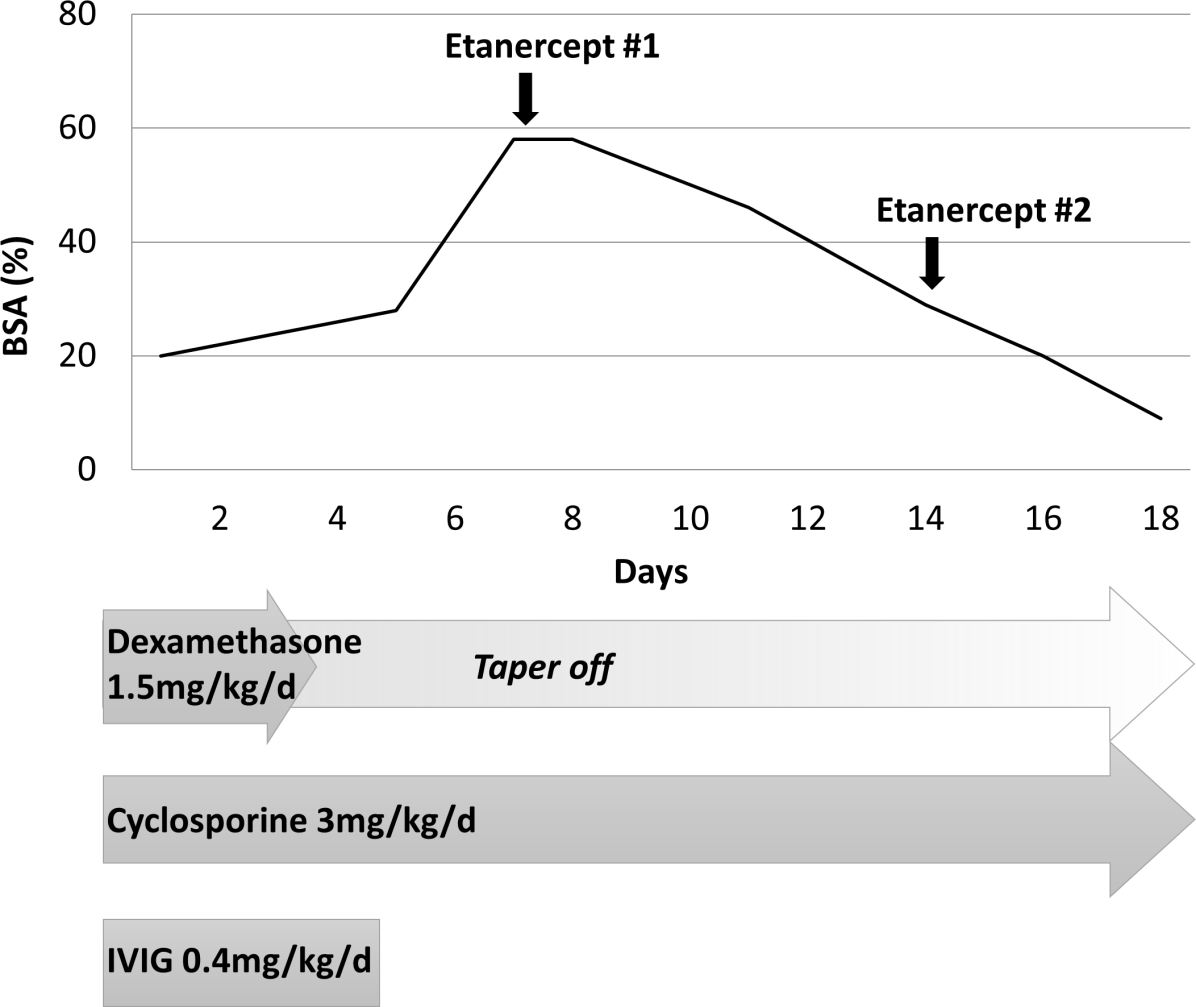
Skin detachment extent according to time. Extent of skin detachment due to toxic epidermal necrolysis during admission. Etanercept (0.8 mg/kg) was injected on hospital day #7 and hospital day #14. BSA, body surface area; HD, hospital day.

Under the diagnosis of SJS/TEN involving 20% of BSA with severity-of-illness score for TEN (SCORTEN) (6) of 1 (tachycardia, heart rate 162), deflazacort was discontinued, and dexamethasone (1.5 mg/kg/day), a 5-day course of IVIG (0.4 mg/kg/day), and cyclosporine 3 mg/kg/day were initiated. The lesions appeared to be stationary for 3 days; however, when IVIG was discontinued on the 6th day of hospitalization, the vesicular lesions progressed to approximately 60% of BSA (**Figures 1A–C**). Etanercept (0.8 mg/kg) was administered subcutaneously (**Figure 2**). After administration, the lesion did not progress, and the bullous lesion also began to epithelialize. However, when re-evaluated on day 15, approximately 30% of the BSA was still involved (**Figures 1D–F**); therefore, a second dose of 0.8 mg/kg of etanercept was administered. Since then, the patient’s condition improved without any complications (**Figure 1G–I**).

## 3 Discussion

TEN is a severe, potentially fatal mucocutaneous disorder (1). Despite the life-threatening nature of TEN, there are currently no standard treatment guidelines for TEN. The mainstay of treatment for TEN involves discontinuation of the culprit drug and provision of supportive care. The efficacy of commonly used systemic therapies, such as systemic steroid therapy, IVIG, and cyclosporine, has not been proven in large randomized trials and has yielded controversial results in smaller studies (6).

Deflazacort, a derivative of prednisolone oxazoline, is commonly used to treat nephrotic syndrome and is associated with low rates of steroid-induced osteoporosis and growth retardation (9). Although steroids are not commonly associated with the risk of SJS/TEN, in our case, deflazacort was the “probable” causative drug according to the ALDEN criteria. While notoriety of deflazacort is not “high risk”, the delay from initial drug intake to index day was “suggestive”, drug was present in the patient at index day, patient had not been exposed to this drug in the past and he had not been introduced to another culprit drug prior to the event (3–5). There have been some prior reports of SJS/TEN caused by deflazacort in literature (9–11). Steroids are classified into four classes based on their cross-reactivity. Deflazacort and corticosteroids are both in class A for cross-reactivity. Therefore, we used dexamethasone, which is in class C (12). Dexamethasone has been used in several cases to treat steroid-induced TEN (11, 13, 14).

While the complete pathogenesis of TEN remains elusive, TEN is understood as a T cell-mediated disease with CD8+ cells causing keratinocyte death with the contribution of granulysin, soluble Fas ligand, and TNF-α (2, 8). Systemic treatments, such as steroids, IVIG, and cyclosporine, inhibit these cytokines; however, their efficacy remains unclear (6, 15, 16). Etanercept is a monoclonal antibody that functions as a TNF-α antagonist by fusing to the extracellular binding domain of the human TNF receptor 2 (7, 17). In preclinical studies, etanercept showed significant immunosuppressive effects by reducing granulysin and TNF-α secretion in blister cells of patients with CTL-mediated severe cutaneous adverse reactions. In a randomized controlled trial comparing etanercept with steroids, etanercept was more effective than systemic corticosteroids in promoting reepithelialization in patients with TEN (8).

However, the use of etanercept in the pediatric population is limited (**Table 1**). In the previously mentioned randomized controlled study (8), the study population was mostly adults with a mean age of 56 years. Six pediatric patients were included in the study, only one of whom was treated with etanercept. Few case reports have described the use of etanercept in pediatric patients with TEN. The predominant causative drug is sulfamethoxazole-trimethoprim, a sulfonamide antibiotic. Treatment protocols varied, with some cases managed solely with etanercept while others concurrently treated with conventional systemic treatments, such as corticosteroids, IVIG, and cyclosporine. In most cases, the etanercept dosage was chosen using a small value of either 0.8 mg/kg/dose or 50 mg. The dosing interval varied from daily to once every 3 days. Physicians are prudent when using etanercept in pediatric patients with TEN due to its off-label application (24).

**Table 1:** Pediatric cases of SJS/TEN treated with etanercept in the literature.

	Age	Sex	Culprit drug	BSA	Onset date (before admission)	Dose of etanercept	Date of administration	Other treatments	Inpatient days	6 months follow-up
This study	44 months	M	Deflazacort	60%	4 days before	0.8 mg/kg/dose	HD 6, 15	IV dexamethasone 1.5 mg/kg for 4 days IVIG 0.4 mg/kg/day for 5 days Cyclosporine 3 mg/kg/day	19	Postinflammatory hyperpigmentation, improving
1 (18)	2 years	M	Ibuprofen or acetaminophen	(–)^*^	(–)^*^	0.4 mg/kg/dose	HD 6, 8	Prednisone 1 mg/kg for 10 days IVIG 1 g/kg/d for 4 days	52	Persistent skin dyspigmentation
2 (19)	11 years	F	SMX/TMP	25%	(–)^*^	25 mg (0.75 mg/kg/dose)	HD 4, 5	IV methylprednisolone 30 mg/kg for 4 days Cyclosporine 5 mg/kg/day for 3 days	(–)^*^	(–)^*^
3 (20)	17 years	M	Carbamazepine	45%	(–)^*^	50 mg (0.8 mg/kg/dose)	HD 1	IV dexamethasone 1 mg/kg for 3 days Cyclosporine 3 mg/kg for 7 days 1.5 mg/kg for 7 days	14	No sequelae
4 (21)	4 years	M	SMX/TMP	12%	4 days before	0.8 mg/kg/dose or 50 mg	HD 1	none	17	Postinflammatory hyperpigmentation, mild atrophic scarring, dry eye disease
5 (21)	7 years	F	acetaminophen or influenza vaccine	50%	4 days before	0.8 mg/kg/dose or 50 mg	HD 1, 3	none	13	Postinflammatory hyperpigmentation, mild atrophic scarring, dry eye disease, trichiasis
6 (21)	18 years	F	SMX/TMP	0%^**^	1 day before	0.8 mg/kg/dose or 50 mg	HD 1	none	4	(–)^*^
7 (21)	7 years	F	amoxicillin-clavulanate or mefenamic acid	10%	1 day before	0.8 mg/kg/dose or 50 mg	HD 1, 2	none	10	Postinflammatory hyperpigmentation, mild atrophic scarring, trichiasis, excessive tearing
8 (22)	13 years	M	SMX/TMP	(–)^*^	2 days before	50 mg	HD 2, 5	IV methylprednisolone 5 mg/kg on HD#2, 2.5 mg/kg q8h for 5 days IVIG 1 g/kg/day for 4 days	13	No sequelae
9 (23)	11 years	M	Unidentified	(–)*	(–)*	25mg	HD 1	Prednisone, IVIG	12	(–)*

BSA, body surface area; HD, hospital day; IVIG, intravenous immunoglobulin; SMP/TMP, sulfamethoxazole-trimethoprim.

^*^Not mentioned. ^**^0% of denudation but 30% of targetoid rash.

In this case, the patient experienced an increase in skin detachment despite conventional systemic treatment. After the administration of etanercept on the 7th day of hospitalization, skin healing commenced, leading to a reduction in the degree of detachment. The patient recovered without complications and was discharged.

This study has limitations in that we have diagnosed TEN due to deflazacort as the probable culprit drug and did not perform skin biopsy to pathologically confirm TEN and *in vivo* or *in vitro* test to confirm the culprit drug. Furthermore, the use of etanercept was not initiated from the onset but rather after other treatments, making it challenging to attribute the observed effects solely to etanercept. Nevertheless, the noticeable improvement in skin lesions following etanercept administration suggests a potentially positive effect of etanercept.

As cases involving the administration of etanercept in pediatric patients are relatively scarce, this study is significant in that etanercept was safely used in children with TEN and led to improvement. Considering the limited number of pediatric patients, this study underscores the importance of further research, including randomized clinical trials, to assess the efficacy and safety of etanercept in the pediatric population.

## Data availability statement

The original contributions presented in the study are included in the article/supplementary material. Further inquiries can be directed to the corresponding author.

## Ethics statement

The studies involving human participants were reviewed and approved by the Seoul National University Hospital Institutional Review Board. (No. 2210-077-1368) Written informed consent to participate in this study was provided by the patient’s parents. Written informed consent was obtained from the minor(s)' legal guardian/next of kin for the publication of any potentially identifiable images or data included in this article.

## Author contributions

MJ: Writing – original draft, Investigation. YC: Investigation, Methodology, Writing – review & editing. YA: Investigation, Writing – review & editing. KL: Writing – review & editing. JP: Writing – review & editing, Conceptualization, Supervision, Writing – original draft. DS: Writing – review & editing.

## References

[1] Duong TA Valeyrie-Allanore L Wolkenstein P Chosidow O. Severe cutaneous adverse reactions to drugs. Lancet (2017) 390(10106):1996–2011. doi: 10.1016/S0140-6736(16)30378-6 28476287

[2] Schwartz RA McDonough PH Lee BW. Toxic epidermal necrolysis: Part I. Introduction, history, classification, clinical features, systemic manifestations, etiology, and immunopathogenesis. J Am Acad Dermatol (2013) 69(2):173.e1–13. doi: 10.1016/j.jaad.2013.05.003 23866878

[3] Roujeau J-C Kelly JP Naldi L Rzany B Stern RS Anderson T. Medication use and the risk of stevens–johnson syndrome or toxic epidermal necrolysis. New Engl J Med (1995) 333(24):1600–8. doi: 10.1056/NEJM199512143332404 7477195

[4] Mockenhaupt M Viboud C Dunant A Naldi L Halevy S Bouwes Bavinck JN. Stevens-Johnson syndrome and toxic epidermal necrolysis: assessment of medication risks with emphasis on recently marketed drugs. The EuroSCAR-study. J Invest Dermatol (2008) 128(1):35–44. doi: 10.1038/sj.jid.5701033 17805350

[5] Sassolas B Haddad C Mockenhaupt M Dunant A Liss Y Bork K. ALDEN, an algorithm for assessment of drug causality in stevens–johnson syndrome and toxic epidermal necrolysis: comparison with case–control analysis. Clin Pharmacol Ther (2010) 88(1):60–8. doi: 10.1038/clpt.2009.252 20375998

[6] Schwartz RA McDonough PH Lee BW. Toxic epidermal necrolysis: Part II. Prognosis, sequelae, diagnosis, differential diagnosis, prevention, and treatment. J Am Acad Dermatol (2013) 69(2):187.e1–16. doi: 10.1016/j.jaad.2013.05.002 23866879

[7] Posadas SJ Padial A Torres MJ Mayorga C Leyva L Sanchez E. Delayed reactions to drugs show levels of perforin, granzyme B, and Fas-L to be related to disease severity. J Allergy Clin Immunol (2002) 109(1):155–61. doi: 10.1067/mai.2002.120563 11799383

[8] Wang CW Yang LY Chen CB Ho HC Hung SI Yang CH. Randomized, controlled trial of TNF-α antagonist in CTL-mediated severe cutaneous adverse reactions. J Clin Invest (2018) 128(3):985–96. doi: 10.1172/JCI93349 PMC5824923 29400697

[9] Jat KR Khairwa A. Deflazacort in comparison to other steroids for nephrotic syndrome. Indian J Nephrol (2012) 22(4):239–45. doi: 10.4103/0971-4065.101238 PMC3495343 23162265

[10] Lee EC Kim GA Koo JW. Toxic epidermal necrolysis associated with deflazacort therapy with nephrotic syndrome. Kidney Res Clin Pract (2014) 33(4):222–5. doi: 10.1016/j.krcp.2014.08.002 PMC4714260 26885481

[11] Youn D Kim M-H Koh SW Kim JW Yoon SE Jeon HK. Toxic epidermal necrolysis induced by deflazacort. Allergy Asthma Respir Dis (2016) 4(3):221–4. doi: 10.4168/aard.2016.4.3.221

[12] Browne F Wilkinson SM. Effective prescribing in steroid allergy: controversies and cross-reactions. Clin Dermatol (2011) 29(3):287–94. doi: 10.1016/j.clindermatol.2010.11.007 21496736

[13] Lauerma AI Reitamo S Maibach HI. Systemic hydrocortisone/cortisol induces allergic skin reactions in presensitized subjects. J Am Acad Dermatol (1991) 24(2 Pt 1):182–5. doi: 10.1016/0190-9622(91)70024-V 1826110

[14] Koh WL Tay YK Koh MJ. Danazol-induced Stevens-Johnson syndrome in a patient with systemic lupus erythematosus. Dermatol Online J (2015) 21(1). doi: 10.5070/D3211025452 25612133

[15] Jacobsen A Olabi B Langley A Beecker J Mutter E Shelley A. Systemic interventions for treatment of Stevens-Johnson syndrome (SJS), toxic epidermal necrolysis (TEN), and SJS/TEN overlap syndrome. Cochrane Database Syst Rev (2022) 3(3):Cd013130. doi: 10.1002/14651858.CD013130.pub2 35274741 PMC8915395

[16] Schneck J Fagot J-P Sekula P Sassolas B Roujeau JC Mockenhaupt M. Effects of treatments on the mortality of Stevens-Johnson syndrome and toxic epidermal necrolysis: A retrospective study on patients included in the prospective EuroSCAR Study. J Am Acad Dermatol (2008) 58(1):33–40. doi: 10.1016/j.jaad.2007.08.039 17919775

[17] Paquet P Paquet F Al Saleh W Reper P Vanderkelen A Piérard GE. Immunoregulatory effector cells in drug-induced toxic epidermal necrolysis. Am J Dermatopathol (2000) 22(5):413–7. doi: 10.1097/00000372-200010000-00005 11048976

[18] Sibbald C Putterman E Micheletti R Treat J Castelo-Soccio L. Retrospective review of drug-induced Stevens-Johnson syndrome and toxic epidermal necrolysis cases at a pediatric tertiary care institution. Pediatr Dermatol (2020) 37(3):461–6. doi: 10.1111/pde.14118 32058621

[19] Gavigan GM Kanigsberg ND Ramien ML. Pediatric stevens-johnson syndrome/toxic epidermal necrolysis halted by etanercept. J Cutan Med Surg (2018) 22(5):514–5. doi: 10.1177/1203475418758989 29421925

[20] Coulombe J Belzile E Duhamel A Rault P Buteau C DeBruycker JJ. Pediatric SJS/TEN subdued by a combination of dexamethasone, cyclosporine, and etanercept. J Cutan Med Surg (2019) 23(5):547–50. doi: 10.1177/1203475419861078 31478770

[21] Eliades P Fonseca M Harp J. Use of etanercept in a series of pediatric patients with stevens-johnson syndrome-toxic epidermal necrolysis spectrum disease. JAMA Dermatol (2020) 156(8):921–2. doi: 10.1001/jamadermatol.2019.3731 32347889

[22] Zander E Hintze TD Sallee B Allen P Miller JL Sagdeo M. Treatment of toxic epidermal necrolysis with etanercept in a pediatric patient. J Pediatr Pharmacol Ther (2021) 26(7):758–61. doi: 10.5863/1551-6776-26.7.758 PMC8475798 34588942

[23] Tian CC Ai XC Ma JC Hu FQ Liu XT Luo YJ. Etanercept treatment of Stevens-Johnson syndrome and toxic epidermal necrolysis. Ann Allergy Asthma Immunol (2022) 129(3):360–365.e1. doi: 10.1016/j.anai.2022.05.009 35598882

[24] Enbrel (etanercept). Thousand Oaks, CA: Immunex Corporation (2023).

